# What Is "Ophthalmic Medicine"?

**Published:** 1915-02-20

**Authors:** 


### WHAT IS "OPHTHALMIC MEDICINE"?

In a recent discussion on the teaching of ophthalmology to medical students one of the speakers—a surgeon on the staff of an ophthalmic hospital—is reported to have advocated special instruction for "those who propose to practise ophthalmic medicine and surgery." The suggestion refers presumably to practitioners who desire to devote their attention as experts to abnormal conditions of the visual apparatus. It is, however, a matter for regret that the proposal is not more explicit. What ophthalmic surgery means we all know, but what is ophthalmic medicine? That certain varieties of nervous, renal, cardio-vascular and other diseases are sometimes attended by symptomatic and structural disturbances in the eyeball and visual tract is no doubt true, but surely this does not mean that such cases require a particular form of medical care which is to be described as "ophthalmic." A case of nephritis remains a case of nephritis whether there are or are not hæmorrhages in the retina; and a similar remark is true of all other general and constitutional diseases which are sometimes accompanied by ocular or visual changes. The true position is, not that patients suffering from any one of these diseases can be divided for purposes of treatment into two groups according as there are or are not ophthalmoscopic or other ocular complications, but rather that all such patients, whether with or without ocular and visual symptoms, need complete medical investigation and treatment; also that the existence of such symptoms makes in this respect no essential difference. To say, as the suggestion with which we are now concerned implies, that while the care of cases of kidney disease not complicated with retinitis is "medicine," while the care of those cases in which retinitis is present is "ophthalmic medicine," is neither scientific classification nor sound practice. It would be equally reasonable to claim that the development of œdema of the eyelids in a case of nephritis is an experience which ought to mean the replacement of the physician by someone who professes to be a specially developed ophthalmic practitioner. The fact is that medicine has no accommodation for these manufactured restrictions and divisions. If a man likes to practise it, let him be content to call himself a physician and be glad if he can prove himself worthy of the name. If, on the other hand, he is a professed surgeon—ophthalmic or otherwise—let him stick to his art and not propose activities of such an artificial order as "ophthalmic medicine." What exactly this term means has yet to be defined. For our own part, to use the "memorable and tremendous words" of Betsey Prig in reference to the fabulous Mrs. Harris, we "don't believe there's no sich a person."

What has just been said by no means implies that there is no real relation between medicine, on the one hand, and local changes in the ocular and visual apparatus on the other. On the contrary, the connection between the two is a very close and important one. In a recent issue of one of the ophthalmic journals we notice that a physician has taken pains to emphasise this relation. More than this, he has had the courage to carry his proposition to its logical conclusion and to insist that the medical relations of ophthalmology ought to be systematically recognised in the teaching of clinical medicine. It is claimed, in other words, that the ophthalmoscope is as much a necessity for the routine investigation of medical cases as is the stethoscope, and that the necessity for this ought to be illustrated in the medical curriculum. In this way, while the local disturbances of the eyeball, and their treatment by local and mechanical measures, would be in the hands of the ophthalmic surgeon, the ocular and visual symptoms which depend on constitutional and visceral disease would be studied in the department of clinical medicine—in association, that is, with the diseases which cause them. There certainly seems much to be said for this view of the question, and it will be of interest to note how such a proposal is received.

